# KCa1.1, a calcium-activated potassium channel subunit alpha 1, is targeted by miR-17-5p and modulates cell migration in malignant pleural mesothelioma

**DOI:** 10.1186/s12943-016-0529-z

**Published:** 2016-06-01

**Authors:** Yuen Yee Cheng, Casey M. Wright, Michaela B. Kirschner, Marissa Williams, Kadir H. Sarun, Vladimir Sytnyk, Iryna Leshchynska, J. James Edelman, Michael P. Vallely, Brian C. McCaughan, Sonja Klebe, Nico van Zandwijk, Ruby C. Y. Lin, Glen Reid

**Affiliations:** Asbestos Diseases Research Institute, Gate 3, Hospital Road, Concord, Sydney, NSW 2139 Australia; Division of Thoracic Surgery, University Hospital Zurich, 8091 Zurich, Switzerland; School of Medicine, University of Sydney, Sydney, NSW 2006 Australia; School of Biotechnology and Biomolecular Sciences, University of New South Wales, Sydney, NSW 2052 Australia; Cardiothoracic Surgical Unit, Royal Prince Alfred Hospital; The Baird Institute and Faculty of Medicine, The University of Sydney, Sydney, NSW 2006 Australia; Sydney Cardiothoracic Surgeons, RPA Medical Centre, Sydney, NSW 2050 Australia; Department of Anatomical Pathology, Flinders Medical Centre, Adelaide, SA 5042 Australia; School of Medical Sciences, University of New South Wales, Sydney, NSW 2052 Australia

**Keywords:** *KCNMA1*, miR-17-5p, Mesothelioma, Therapeutic targets, KCa1.1, microRNA, Integrative analysis

## Abstract

**Background:**

Malignant pleural mesothelioma (MPM) is an aggressive, locally invasive, cancer elicited by asbestos exposure and almost invariably a fatal diagnosis. To date, we are one of the leading laboratory that compared microRNA expression profiles in MPM and normal mesothelium samples in order to identify dysregulated microRNAs with functional roles in mesothelioma. We interrogated a significant collection of MPM tumors and normal pleural samples in our biobank in search for novel therapeutic targets.

**Methods:**

Utilizing mRNA-microRNA correlations based on differential gene expression using Gene Set Enrichment Analysis (GSEA), we systematically combined publicly available gene expression datasets with our own MPM data in order to identify candidate targets for MPM therapy.

**Results:**

We identified enrichment of target binding sites for the miR-17 and miR-30 families in both MPM tumors and cell lines. RT-qPCR revealed that members of both families were significantly downregulated in MPM tumors and cell lines. Interestingly, lower expression of miR-17-5p (*P =* 0.022) and miR-20a-5p (*P =* 0.026) was clearly associated with epithelioid histology. We interrogated the predicted targets of these differentially expressed microRNA families in MPM cell lines, and identified KCa1.1, a calcium-activated potassium channel subunit alpha 1 encoded by the *KCNMA1* gene, as a target of miR-17-5p. KCa1.1 was overexpressed in MPM cells compared to the (normal) mesothelial line MeT-5A, and was also upregulated in patient tumor samples compared to normal mesothelium. Transfection of MPM cells with a miR-17-5p mimic or *KCNMA1*-specific siRNAs reduced mRNA expression of KCa1.1 and inhibited MPM cell migration. Similarly, treatment with paxilline, a small molecule inhibitor of KCa1.1, resulted in suppression of MPM cell migration.

**Conclusion:**

These functional data implicating KCa1.1 in MPM cell migration support our integrative approach using MPM gene expression datasets to identify novel and potentially druggable targets.

**Electronic supplementary material:**

The online version of this article (doi:10.1186/s12943-016-0529-z) contains supplementary material, which is available to authorized users.

## Background

Malignant pleural mesothelioma (MPM) is an aggressive tumor occurring in the lining of the lungs, induced by exposure to asbestos. MPM has poor prognosis, and palliative chemotherapy is often the only treatment modality that can be offered [1, 2]. Since the adoption of cisplatin and pemetrexed as the standard of care [3] a decade ago, there has been little therapeutic progress and the identification of new therapeutic targets for MPM is an urgent unmet need.

Genome-wide gene expression profiling studies using microarray and next generation sequencing (NGS) have facilitated identification of disease-specific expression profiles and many are publicly available. While most studies have focused on the identification of a single therapeutic candidate, the greatest challenge remains the interpretation of data within the context of cancer cell biology. Bioinformatic tools such as Gene Set Enrichment Analysis (GSEA) [4] have improved interpretation of microarray and NGS data for downstream functional validation. In MPM, gene expression profiling studies have identified several novel targets including *MMP14* [5], *ALCAM* [6], *NME2*, *CRI1, PDGFC* and *GSN* [7]. However, there was little commonality between these studies, and to date no pharmaceutical approach to targeting these candidates has been developed. At a systems level, pathway analysis has revealed enrichment of genes in MPM belonging to cellular processes such as cellular metabolism, cytoskeletal re-organization, apoptosis, spindle checkpoint and cell cycle progression and regulation [5, 8, 9]. Many of these pathways, however, have not been explored in detail.

Since MPM is characterized by alterations in multiple genes, we hypothesized that a strategy to inhibit and/or restore a single target gene is unlikely to be effective. In comparison, new insights into the involvement of microRNAs in the regulation of MPM growth [10] have provided an alternative way to inhibit MPM growth with the potential to be successfully translated into a new therapeutic approach for MPM [11]. MicroRNAs are small non-coding RNAs involved in post-transcriptional control of gene expression [12]. They form a complex network where each microRNA regulates multiple mRNAs and each mRNA is regulated by multiple microRNAs. Changes in microRNA expression are associated with proliferation and drug resistance of cancer cells, and microRNAs can act as oncogenes or tumor suppressors [13–16]. Making use of data from our previous studies [17–19], we present here an integrative approach by comparing microRNA and mRNA gene expression datasets to identify enriched biological themes that can be translated into potential druggable targets for MPM, as well as functional data revealing that KCa1.1 is a potential therapeutic target in MPM.

## Results and discussion

### Identification of target binding site of differentially expressed genes in MPM cell lines and tumors (enriched microRNA binding sites)

**Fig. 1:**
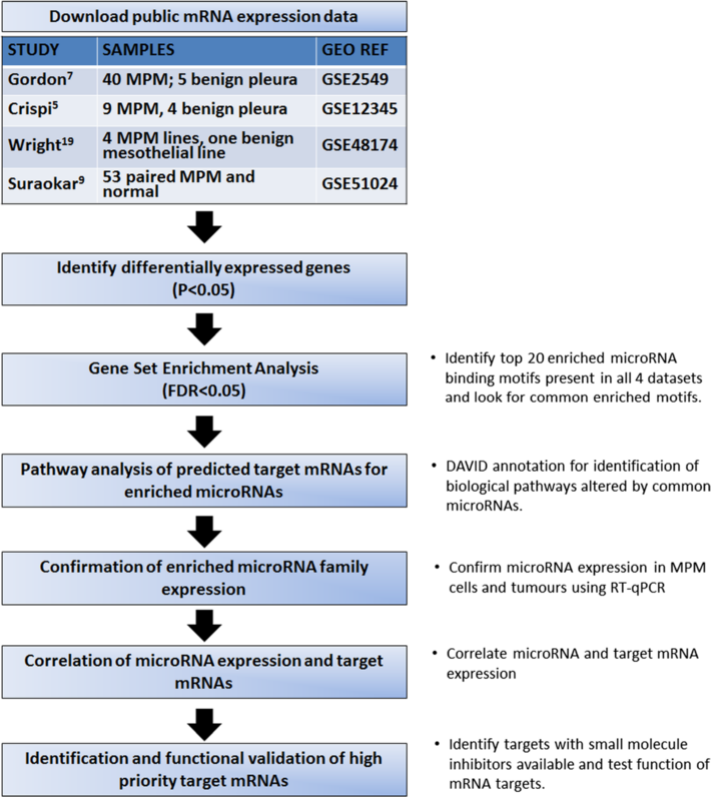
Analysis pipeline. Differentially expressed gene lists in MPM from four public datasets (*P* < 0.05) were subjected to GSEA analysis using the C3 list in the Molecular Signatures Database (MSigDB v4.0) to identify enriched 3’UTR microRNA binding motifs [4]. The top 20 enriched microRNA motifs identified at *P* < 0.05 (False discovery rate adjusted) were considered significant and ranked between these four studies. Predicted target mRNAs from these enriched microRNA families were analyzed further using Gene Ontology (DAVID [68]) and Pathway Enrichment (Partek Genome Suite) to elucidate affected regulatory pathways. Confirmation of dysregulated candidate microRNA families in MPM cell lines (*n* = 7) and patient tumors (*n* = 59) were carried out using RT-qPCR. Furthermore, correlation of enriched microRNAs (downregulated) to gene expression of predicted targets (upregulated) were extracted based on our previous published array dataset [19]. Thus this analysis pipeline identifies and ranks candidate targets according to significance *P* value, correlation between miRNA-mRNA array data as well as being able to be targeted functionally by small molecule inhibitors

MPM is a complex disease driven by polygenic dysregulation and we hypothesized that an integrated microRNA-mRNA approach would assist us in identifying dysregulated layers of gene regulation affected by microRNAs. Their gene targets, in turn, can potentially serve as therapeutic targets. Previous studies have identified extensive changes in microRNA expression in MPM, as recently reviewed [10]. We have profiled gene expression in MPM cell lines compared to MeT-5A (immortalized normal mesothelial cell line) [19], and have demonstrated up and down regulation in multiple microRNAs in MPM patient tumor samples and cell lines [17, 18, 20]. To our knowledge, we are one of the few laboratories in the world who have studied microRNA expression profiles in MPM tumor and normal mesothelium samples in order to identify dysregulated microRNAs playing an important functional role in the biology of MPM. Therefore, we systematically interrogated 1319 differentially expressed mRNAs (*P* <0.05) in our dataset [19] using the Molecular Signatures Database (MSigDB) [4]. This led to the identification of enriched microRNA binding motifs, i.e., miR-30, miR-15 and miR-17 (Fig. 1). We then applied this GSEA strategy [4] to the three remaining MPM gene expression datasets [GSE2549, GSE12345, GSE51024] (outlined in Fig. 1 and Additional file 1: Table S1) to identify commonly enriched microRNA families.

### Binding sites for miR-17 and miR-30 microRNA families are enriched in all gene expression datasets

**Fig. 2:**
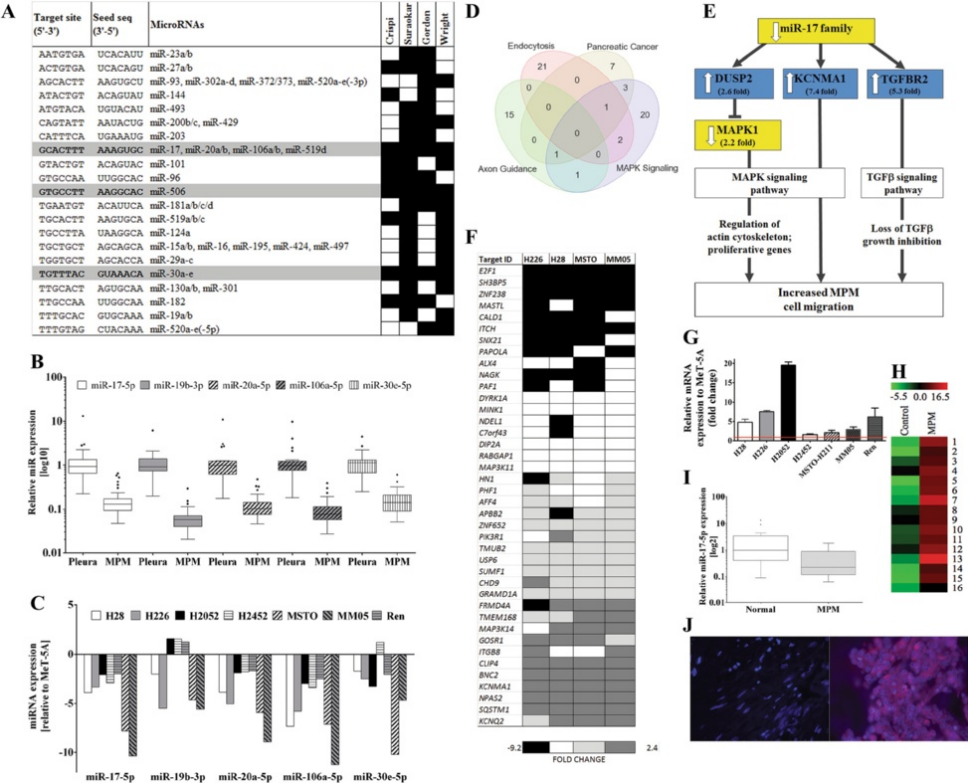
Identification of enriched microRNA families. **a** The top 20 enriched microRNA binding motifs in each MPM gene expression study were compared and the overlap between studies identified. Families enriched in more than one dataset are included in the table (see Additional file 1: Table S1 for top 20 enriched families in each dataset). RT-qPCR confirmed decreased expression of the miR-17 family in (**b**) MPM tumors (*n* = 59) compared with normal pleural tissue (*n* = 22) and (**c**) MPM cells lines compared with MeT-5A. The formalin-fixed paraffin embedded (FFPE) tumor tissues used in this study were described previously [62]. Total RNA was extracted from cell lines, tumors and normal pleura and used as template in RT-qPCR using microRNA-specific TaqMan assays (Additional file 1: Table S3) as previously described [17, 18]. Relative expression levels were calculated using the 2^-ΔΔCq^ method [63] relative to MeT-5A or normal pleura. **d** Analysis of the top four enriched pathways related to the miR-17 family identified a number of target genes involved in multiple pathways. **e** Key miR-17 family target genes are coordinately regulated in signaling pathways contributing to MPM cell migration. Blue denotes upregulation > 1.5 fold, Yellow denotes downregulation < 1.5 fold. White arrow denotes direction in change of expression using data from Wright *et al.* [19]. **f** Expression analysis identified 40 predicted targets of miR-17 that were differentially expressed between MPM cells and MeT-5A cells; 20 of these targets were upregulated, including *KCNMA1* and RT-qPCR confirmed upregulation of *KCNMA1* in MPM cell lines (**g**). In a second series of tumor samples consisting of fresh-frozen samples from extrapleural pneumonectomy (EPP) patients, *KCNMA1* was upregulated (**h**) and miR-17-5p downregulated (**i**) compared with normal pleural tissue controls (see Additional file 1: Table S4 for patient characteristics). **j** KCa1.1 expression in MPM tumor samples were analyzed by immunofluorescence microscope (Objective 40×, Axio imager.M2) showed high level of KCal.1 expression (*right*) of tumor area and low to no KCa1.1 expression of the non-tumor area (*left*)

Two families, miR-17 and miR-30, were identified amongst the top 20 enriched microRNA families across the four datasets (Fig. 2a). The miR-17 family includes miR-20a/b, miR-93 and miR-106a/b and forms clusters with members of the miR-18, miR-19 and miR-25 families. The miR17 ~ 92 cluster, located on chromosome 13 consists of miR-17, miR-18a, miR-19a, miR-20a, miR-19b-1 and miR-92a-1, with two paralogues; the miR-106b ~ 25 (miR-106b, miR-93 and miR-25) and miR-106a ~ 363 (miR-106a, miR-18b, miR-20b, miR-19b-2, miR-92a-2 and miR-363) clusters. The miR-17 ~ 92 cluster has been characterized as oncogenic in various solid and hematological malignancies (reviewed in [21]), but intriguingly there is frequent copy number loss or deletion of the genetic locus at 13q31 [22] in various cancers and downregulation of this cluster is also implicated in aging [23]. Downregulation of the miR-30 family (consisting of miR-30a, miR-30b, miR-30c, miR-30d and miR-30e) has also been associated with various malignancies including colorectal [24], gastric [25], lung [26] and thyroid cancers [27]. Interestingly, increasing the levels of miR-17 was shown to inhibit breast cancer cell growth [16], while miR-30a has been found to suppress migration and invasion of breast cancer cells [28], and proliferation of colon [24] and hepatocellular carcinoma cells [29]. In addition, both miR17 and miR-30 families are predicted to target many cancer related genes, and have critical roles in cell cycle, apoptosis, migration and proliferation. Since both microRNA families are significantly enriched in our integrative analysis, this implicates their important roles in cancer biology including mesothelioma.

### The miR-17 family is consistently downregulated in MPM and its predicted targets are associated with multiple cancer-related pathways

We validated microRNA expression using RT-qPCR and found consistent downregulation of multiple miR-17 family members and miR-30e in MPM in a set of 23 normal pleura and 60 formalin-fixed, paraffin-embedded (FFPE) tumor samples from our biobank, as well as a panel of 7 MPM and mesothelial cell lines (Fig. 2b and c). Lower expression of miR-17-5p (*P =* 0.022) and miR-20a-5p (*P =* 0.026) was significantly associated with epithelioid histology, as was the co-expressed miR-19b-3p (*P =* 0.016). Since miR-30e-5p expression did not show any association with clinical characteristics in MPM, we focused further attention on the miR-17 family.

Previous studies addressing the role of miR-17 in cancer biology have shown this to be complex. While miR-17 expression is frequently reported to be upregulated as part of the miR-17 ~ 92 cluster [21], miR-17-5p downregulation and/or loss of heterozygosity/gene deletion at 13q31 has been reported in various tumors [22]. Thus the role of these microRNAs is likely to be context specific. Pathway enrichment analysis revealed that the predicted targets of miR-17 family are enriched in several key cancer-related signaling pathways previously implicated in MPM biology and treatment, including MAPK signaling [30], ErbB signaling [31], Focal adhesion [32], TNF signaling [33] and TGF-beta signaling pathways [34] (Additional file 1: Table S2). Furthermore, the top four enriched pathways share genes linked to MPM cell migration, in particular *MAPK1*, *P* = 0.03, −2.2 fold in MPM (MAPK, Axon Guidance, Pancreatic Cancer pathways) and *TGFBR2*, *P* = 0.0002, > 5 fold in MPM (Pancreatic Cancer, MAPK, Endocytosis pathways) (Fig. 2d). Specifically, key target genes of the miR-17 family are coordinately regulated in signaling pathways contributing to MPM cell migration (Fig. 2e), where downregulation of miR-17 is associated with increased *DUSP2* expression, an inhibitor of MAPK1. Interestingly, *DUSP2* has been shown to be downregulated by the miR-17 family member miR-20a [15]. Decreased miR-17 also leads to increased *KCNMA1* expression (which in turn interacts with the MAPK signaling pathway [35], and increased *TGFBR2* [36] and altered TGFβ2 signaling. These gene expression changes indicate that all these pathways together contribute to an increase in MPM cell migration (Fig. 2d and e), and collectively these results implicate a broad impact of the miR-17 family in MPM biology.

### Reduced miR-17-5p expression is correlated with increased expression of *KCNMA1*

We investigated the correlation between miR-17-5p expression and mRNA expression of its predicted targets from the same MPM cell lines [19] and identified that 20 out of 40 target genes demonstrated the expected inverse relationship between miRNA and mRNA levels, *P* <0.05 (Fig. 2f). Prioritizing these gene targets based firstly on those that showed significant differential gene expression (>5-fold change) and secondly on the availability of small molecule inhibitors/drugs, led to identification of *KCNMA1*. This gene encodes the calcium-activated potassium channel subunit alpha 1, KCa1.1 previously reported to be upregulated in prostate [37], breast [38] and other cancers [39, 40]. RT-qPCR confirmed upregulation of *KCNMA1* mRNA in MPM cell lines compared to the normal immortalized mesothelial line MeT-5A (Fig. 2g), which was associated with decreased expression of miR-17-5p (Fig. 2c). We further analyzed *KCNMA1* gene expression in fresh-frozen samples of normal pleura and MPM from our biobank. In common with other tumor types, we observed increased expression of *KCNMA1* (Fig. 2h) in MPM tumor samples as well as downregulation of miR-17-5p (Fig. 2i). Immunofluorescence studies further indicated that MPM tumor samples with high tumor cell content expressed high levels of KCal.1 (Fig. 2j right); in contrast, no expression was detected in areas without tumor cells (Fig. 2j left).

Potassium ion channels such as KCa1.1 are the subject of increasing interest in cancer research due to their observed effects on cell processes including cell proliferation, cell adhesion, angiogenesis, cell migration and metastasis (reviewed in Pardo [41]). For example, expression of Kv10.1 channel (EAG1, product of the *KCNH1* gene) is normally limited to the brain, but it is overexpressed in many tumor types and knockdown in overexpressing cells was found to reduce viability [42]. Furthermore, there is evidence that altered microRNA expression is involved in the regulation of potassium channels in cancer. In glioblastoma [43] for example, increased EAG1 protein expression was associated with decreased miR-296-3p levels and in osteosarcoma [44], increased EAG1 is correlated with reduced miR-34a. Kv11.1 (HERG) is upregulated in pancreatic cancer as a consequence of miR-96 downregulation [45] and miR-211 expression is inversely correlated with KCa1.1 in melanoma [39]. In each case, ectopic expression of these microRNAs reduced channel expression and inhibited proliferation of the cancer cells.

### *KCNMA1* gene is a direct target of miR-17-5p and genetic and pharmacological inhibition of its protein, KCa1.1 modulates migration in MPM cells

**Fig. 3:**
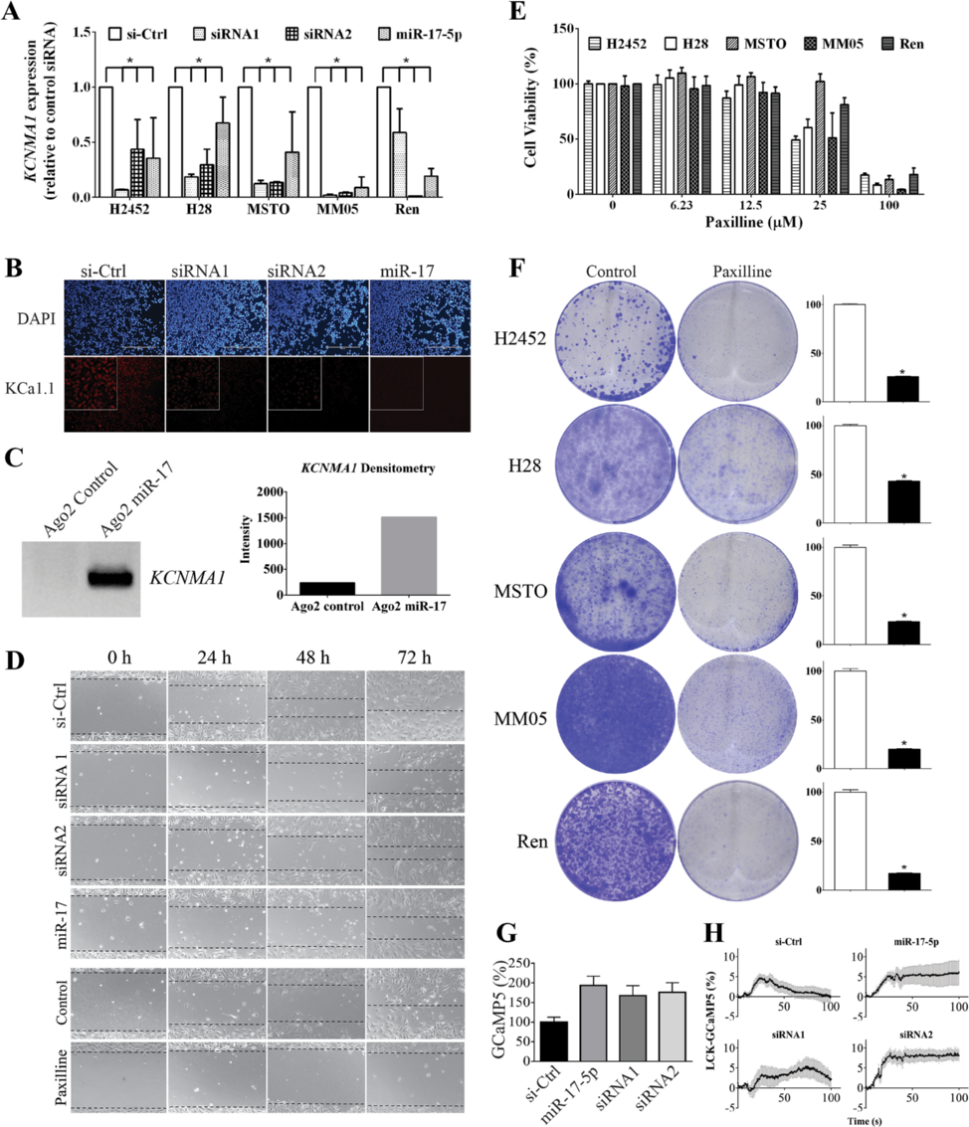
Molecular and pharmacological inhibition of KCa1.1 inhibits MPM cell line migration. **a** Transfection with miR-17-5p mimic or *KCNMA1*-specific siRNA (10 nM) resulted in significantly (* = *P* < 0.01) decreased *KCNMA1* mRNA gene expression in MPM cell lines when compared with controls (Individual *P* values are included in Additional file 1: Table S5). RT-qPCR carried out as described for Fig. 2. **b** Immunofluorescent staining of KCa1.1 (Rabbit anti-KCNMA1, 1:500, Sigma) showed significant reduction in expression of KCa1.1 protein in MSTO cells transfected with miR-17-5p mimic or *KCNMA1*-specific siRNA (final concentration of 10 nM; bar = 400 μm). **c** Levels of *KCNMA1* were measured following AGO2-IP using PCR and were higher in cells transfected with the miR-17 mimic. **d** Transfecting with miR-17 mimic did inhibit migration of mitotically inactivated H28 cells. Similar results obtained with other MPM cell lines are presented in Additional file 1: Figure S5. **e** In proliferation assays, the growth of MPM cell lines was inhibited by high concentrations of the KCa1.1 blocker paxilline. In contrast, a sub-lethal dose of paxilline (12.5 μM) inhibited migration (D, last 2 rows) and colony forming ability of MPM cells, plated at low density (**f**, histograms represent total dye in lysed colonies, as a percentage normalized to control, * *P* value all < 0.0001). *P* values for each comparisons are individually presented in the Additional file 1: Table S5. **g** Levels of cytosolic Ca^2+^ were estimated by overexpressing the soluble GCaMP5 Ca^2+^ reporter in MPM cells co-transfected with siRNA. Note, that the fluorescence intensity of the reporter is increased after the KCNMA1 knockdown, * *P* < 0.05, ANOVA with Holm-Sidak’s multiple comparisons test. **h** Analysis of the Ca^2+^ influx in MPM cells overexpressing the membrane-targeted LCK-GCaMP5 Ca^2+^ reporter and co-transfected with siRNA or miR-17-5p mimic. Calcium influx was induced at 5 s after the start of recording by application of 90 mM K^+^-containing buffer. Graphs show mean ± SEM fluorescence intensity of the reporter

To determine whether miR-17-5p has a direct effect on modulating *KCNMA1* expression, we transfected MPM cells with a miR-17-5p mimic. Increasing levels of miR-17-5p resulted in a decrease in *KCNMA1* (Fig. 3a) and *TGFBR2* (Additional file 1: Figure S1) mRNA expression in MPM cells, similar to that found with MPM cells treated with *KCNMA1*-specific siRNA (Fig. 3a). Furthermore, transfection with either miR-17-5p mimic or siRNAs designed to target the *KCNMA1* transcript, significantly reduced expression of KCa1.1 protein, as seen by the reduced immunofluorescent staining of the membrane in transfected MSTO cells (Fig. 3b). To confirm the interaction between miR-17-5p and the 3’UTR of *KCNMA1*, we used AGO2 immunoprecipitation [46, 47] to isolate the AGO2 protein and associated RNA following transfection with miR-17-5p mimic. This resulted in a clear increase in the *KCNMA1*-specific RT-PCR signal after transfection with miR-17-5p mimic, indicating that miR-17-5p directly interacts with *KCNMA1* in MPM cells (Fig. 3c).

Pathway analysis shows that *KCNMA1* is also involved in a range of cellular processes, including Axon Guidance, Focal Adhesion and Wnt Signaling, so we next investigated the effects of inhibiting KCa1.1 in MPM cells. Silencing *KCNMA1* expression with siRNAs or miR-17-5p mimics had limited effects on the proliferation of a panel of MPM cell lines (Additional file 1: Figure S2), and did not lead to changes in the cell cycle (Additional file 1: Figure S3) or induction of apoptosis (Additional file 1: Figure S4) in transfected cells. This is in contrast to the inhibition of breast cancer [38] and melanoma [39] cell lines following *KCNMA1* knockdown. Both miR-17-5p and *KCNMA1*-specific siRNAs, however reduced migration in H28 (Fig. 3d) and other MPM cell lines (Additional file 1 Figure S5), and the invasive capacity of cells in a modified agarose spot assay (Additional file 1: Figure S6). Similarly, modulation of KCa1.1 activity with the inhibitor paxilline suppressed migration of MPM cell lines. Paxilline inhibited cell proliferation at concentrations greater than 25 μM (Fig. 3e) and treating MPM cells at a sub-lethal dose (12.5 μM) resulted in significant inhibition of migration (Fig. 3d) and cell colony formation (Fig. 3f). Interestingly, MPM cell lines were less sensitive than breast or prostate cancer lines, in which growth was inhibited by 15 μM paxilline [38]. These data are consistent with previous results implicating a role in cell migration for calcium-activated potassium channels in general [41] and that KCa1.1 in particular, has been shown to be important in the migration of glioma cells [48], invasiveness of melanoma cells [39], and transendothelial migration in metastatic breast cancer [48, 49].

Cell migration and invasion is dependent on calcium flux, which contributes to changes in cellular volume [50, 51]. The blocker-sensitive outward potassium current generated by large conductance potassium channels such as KCa1.1 can functionally couple with other ion channels to control calcium flux, thus facilitating migration and invasion by regulating cell volume [52]. In MPM cells, KCNMA1 knockdown led to an increase in the basal intracellular calcium concentration (Fig. 3g). As KCNMA1 is the major contributor to the resting membrane potential, this is likely due to a partial membrane depolarization and higher activity of Ca^2+^ channels [53, 54]. Furthermore, KCNMA1 silencing resulted in a more prolonged elevation in submembrane Ca^2+^ levels in response to high K^+^ containing buffer (Fig. 3h). Together, these experiments demonstrate a major impact of KCNMA1 silencing on Ca^2+^ handling by MPM cells. In light of these observations it is interesting that changes in basal Ca^2+^ levels following KCNMA1 knockdown had negligible effects on cell growth, despite altered Ca^2+^ handling being linked to apoptosis resistance in MPM cells [55]. Further research is required to understand exactly how the loss of KCNMA1 activity and resultant changes in Ca^2+^ flux reduces migration and invasion without impacting apoptosis, and is beyond the scope of the current study.

Potassium channels have been implicated in drug resistance of cancer cells. One example is provided by the miR-296-3p-mediated downregulation of EAG1 leading to reversal of anticancer drug resistance in glioma cells [43]. There is conflicting evidence, however, for the role of potassium channels in the toxicity of cisplatin, a platinum-based drug frequently used in chemotherapy for MPM. Increased expression of the potassium channel Kv10.1 (EAG, *KCNH1*) was linked to the drug resistance of glioblastoma cells [43], whereas inhibition of the elevated levels of Kv11.1 (HERG, *KCNH2*) induced by cisplatin selection had no effect on the toxicity of cisplatin in epidermal or liver cancer cells. In contrast, it was found to be essential for cisplatin-induced apoptosis in gastric cancer [56]. We did not, however, observe sensitization of MPM to either cisplatin or gemcitabine in the presence of paxilline (Additional file 1: Figure S7), suggesting that KCa1.1 is not involved in the cytotoxic activity of these drugs in MPM, and is not solely responsible for the potassium flux modulated by amphotericin B and bumetanide previously observed in MPM [57].

### Therapeutic targeting of potassium channels in cancer

Growing evidence suggests that the potassium channels Kv10.1, Kv11.1, KCa1.1 and KCa3.1 have important roles in cancer cell invasion and metastasis [38, 41], and as MPM is a locally invasive cancer with high migratory capacity, that targeting these channels is a potential therapeutic option. Kv11.1 (hERG, encoded by the *KCNH2* gene) is overexpressed in many cancers [41] and our MPM cell lines (1.78-fold upregulation, *P* = 0.03). First linked to cancer through the finding that resting membrane potential in neuroblastoma cells was linked to proliferation, Kv11.1 has since been found to be expressed at high levels in multiple tumor types [41]. While this potentially makes Kv11.1 a good target, it is also expressed in the heart where it is related to long QT syndrome, making it difficult to envisage a cancer-specific therapeutic window for Kv11.1 inhibition. The Kv10.1 (EAG1, product of the *KCNH1* gene) channel is normally only expressed in the brain, but is upregulated in multiple cancer types [41], and its inhibition or knockdown reduces cell migration and viability [42]. The important role of this channel in cancer was demonstrated by its ability to confer invasive growth characteristics to otherwise non-tumorigenic cell lines in xenograft models [41]. However, its close evolutionary relationship to Kv11.1 reduces the possibility that drugs targeting Kv10.1 will be sufficiently specific.

Other potassium channels may represent preferable targets to control cancer cell migration. For example, inhibition of KCa2.3 reduced bone metastases in a model of breast cancer [58], and blocking KCa1.1, which was induced by ionizing radiation, inhibited the increase in glioma cell migration [59]. Targeting KCa1.1 in MPM could be achieved using a channel blocker such as paxilline. While an inhibitory dose may be difficult to reach via systemic inhibition, the typical restriction of MPM tumor growth to the pleural cavity provides an alternative loco-regional route for delivery of paxilline or other potassium channel blockers. Alternatively, the post-transcriptional regulation of *KCNMA1* by miR-17-5p suggests that KCa1.1 could be an important target of a miR-17-5p mimic-based therapy.

## Conclusions

We have shown here that an integrated approach, combining publicly available gene expression datasets, is an effective and practical strategy to identify therapeutic targets in MPM. These targets can be both proteins that are inhibited via traditional pharmacological interventions, as well as microRNAs themselves, as illustrated by a near-complete response in a MPM patient failing on standard chemotherapy, who received a miR-16 mimic packaged in (targeted) nanocells [11], revealing that microRNA-based treatment concepts are valid [18]. We have demonstrated that KCa1.1 is a direct target of miR-17-5p and inhibition of this combination modulates cell migration in MPM.

## Methods

### Cell lines

Human mesothelioma cell lines H28, H226, H2052, H2452, MSTO and the immortalized normal mesothelial line MeT-5A were obtained from the American Type Cell Culture repository (ATCC, Rockville, USA) and MM05 [60] and REN [61] were described previously and kindly provided by collaborators. All cells were grown in RPMI with 10 % fetal bovine serum (FBS) at 37 °C with 5 % CO_2_. REN cells were grown in Ham’s F12 medium. All medium and FBS were obtained from Life Technologies or Sigma.

### Tissue samples

Formalin-fixed paraffin embedded (FFPE) tumor tissues used in this study were part of a reported series of extrapleural pneumonectomy (EPP) patients collected between 1994 and 2009 from the Royal Prince Alfred Hospital (RPAH) or Strathfield Private Hospital, Sydney [62]. Waiver of consent for the use of these archival samples was granted by the Human Research Ethics Committee (HREC) at Concord Repatriation General Hospital (CRGH), Sydney, Australia (CH62/6/2009/078). Demographics for this population have been described elsewhere [18]. For the fresh-frozen tumor samples and normal pleural tissue samples, all patients gave informed written consent and the project was approved by the HREC at CRGH (HREC/11/CRGH/75) and RPAH (HREC/10/RPAH/599).

### RNA isolation

Total RNA was extracted from cell lines and fresh-frozen tissue samples using the TRIzol reagent (Life Technologies, Carlsbad, CA) and from FFPE tumors and normal pleura using the RNeasy FFPE kit (Qiagen, Hilden, Germany). RNA was quantified using a Nanophotometer (Implen, Munich, Germany) and quality was assessed using an Agilent 2100 Bioanalyzer (Agilent Technologies, Santa Clara, CA). Samples with RNA integrity numbers (RINs) >8.0 were used for microarray analysis.

### Microarray and TaqMan Low Density Array (TLDA) data acquisition

Microarray profiling experiments were performed using NCode Human Non-coding RNA microarrays (Life Technologies) according to MIAME guidelines, as previously described [19]. All expression data is available at the National Centre for Biotechnology Information Gene Expression Omnibus [GSE48174]. TaqMan Low Density Array (TLDA) profiling was performed as previously described [18], following the profiling protocol without pre-amplification. TLDAs were run on a Viia7 Real-Time machine (Life Technologies). Data was analyzed using the 2^-∆∆Cq^ method [63], with normalization to the average RNU6B Cq, and calculation of expression values was made relative to MeT-5A. MicroRNAs with Cq values >35 were excluded from analysis.

### Quantitative RT-PCR in real time (RT-qPCR)

MicroRNA and mRNA expression levels from microarray analysis were validated using RT-qPCR in the five cell lines assayed using NCode and TLDA arrays. MicroRNA expression levels were also validated in fresh-frozen and FFPE tumor specimens from MPM patients and samples of normal mesothelium using TaqMan microRNA assays (see Additional file 1: Table S3 for assay IDs). Primers were designed for mRNA targets using the Universal Probe Library (UPL) algorithm provided by Roche (Additional file 1: Table S3). To quantify mRNA expression, total RNA (250 ng for cell lines) was reverse transcribed to cDNA using the Superscript III cDNA synthesis kit (Life Technologies) using oligo-dT primers (100 ng/μL). After reverse transcription, cDNA was diluted 1:5 with 2 μL of this product used as template in real-time qPCR. All reactions were run in triplicate on a Viia7 real-time machine (Life Technologies), using KAPA SYBR Fast qPCR master mix (Kapa Biosystems). All reactions had an initial enzyme inactivation step at 95 °C for 10 min followed by 40 cycles of 95 °C for 15 s and 55 °C for 30 s. 18S ribosomal RNA was used as the reference for qPCR data normalization and no template and no-RT samples included as negative controls. For microRNA expression, 100 ng of total RNA was reverse transcribed using pooled microRNA specific primers and the MicroRNA Reverse Transcription Kit (Life Technologies) as previously described [64]. Specific TaqMan assays were used to amplify 10 ng cDNA using the KAPA Probe FAST qPCR master mix. Relative expression levels were calculated using the 2^-ΔΔCq^ method as described [63] with MeT-5A designated a value of 1 (all fold-change values were calculated relative to this cell line). Genes were deemed technically replicated if the direction of expression was consistent with microarray data, and the magnitude of change was greater than 2-fold.

### RNA interference (RNAi), paxilline treatment and growth assays

Knockdown of *KCNMA1* was performed using two previously published siRNA sequences [37] and reintroduction of target microRNAs was performed using microRNA mimics. All siRNAs and microRNA mimics were obtained from Shanghai GenePharma. MPM cells were reverse transfected as described previously [65]. Briefly, 2,500 cells were reverse transfected with varying concentrations of microRNA mimic or siRNA using Lipofectamine RNAiMAX (Life Technologies) at 0.1 μL per well. RNA was extracted 48 h after transfection to confirm knockdown efficiency using RT-qPCR. Cell growth assays were performed over a 5 d period to monitor changes in cell proliferation as described previously [18]. Briefly, at the indicated time points, medium was aspirated from replicate plates, which were then frozen at −80 °C. At the conclusion of the experiments, relative cell numbers were determined by staining with 200 μL/well of SYBR Green I (Life Technologies) 1:4000 in a hypotonic lysis buffer (10 mM Tris HCl (pH 8), 5 mM EDTA, 0.1 % Triton X-100) overnight in the dark at 4 °C and then quantified by fluorimetry, measured using a FLUOstar Optima (BMG LabTech, Ortenberg, Germany) set to 485 nm excitation and 535 nm emission. Fluorescence intensity in siRNA or mimic-transfected cells was normalized to control-transfected cells. Each experiment was performed in triplicate. Growth inhibition using the small molecule inhibitor paxilline (Sigma Aldrich), in the presence or absence of cisplatin or gemcitabine was carried out using the same assay.

### AGO2 Immunoprecipitation (AGO2-IP)

Cells were transfected with miR-17-5p or control mimic and AGO2 protein was immunoprecipitated as previously described [46, 47]. Total RNA isolation was carried out using TRIzol reagent, followed by RT-PCR with primers specific for *KCNMA1* mRNA. PCR products were run on 2 % agar gel and visualized following ethidium bromide staining. Imaging was carried out using a Gel Logic 2200 Imaging System (Kodak) under non-saturating conditions. Densitometry of band intensity was carried out using the same software.

### Immunofluorescence staining of KCa1.1 protein expression

Cells transfected with *KCNMA1*-specific siRNA, miR-17-5p mimic or control were fixed in paraformaldehyde solution (4 % v/v in PBS, Sigma, St. Louis, MO, USA) for 15 min, washed three times with PBS and permeabilized with 0.2 % Triton X-100 in PBS for 5 min. Fixed cells were blocked with PBS containing 0.1 % Triton and 10 % fetal bovine serum for 1 h at room temperature. For immunostaining, cells were incubated for 2 h at room temperature with 20 μg/mL rabbit anti-KCa1.1 antibody (Sigma) in blocking solution. After three washes with PBS, cells were incubated for 1 h at room temperature with an AlexaFluor 488-labeled goat anti-rabbit antibody (Life Technologies). Nuclear counterstaining was performed with 0.5 μg/mL DAPI. Immunostained cells were imaged with an EVOS fluorescence cell imaging system (Life Technologies).

### Colony formation assay

Cells were plated in triplicate in 96-well plates at 2500 cells/well and transferred to 6-well plates 24 h post transfection. After incubation for 10–14 d at 37 °C, cells were fixed with 70 % ethanol and stained with 0.1 % Crystal Violet. The plates were then de-stained with 2 % SDS and absorbance was measured at 562 nM using a FLUOstar Optima.

### Migration assay

MPM cell migration was measured using a scratch (or wound-healing) assay. Briefly, transfected cell were plated in 24-well plates and 24 h post-transfection 10 μg/mL Mitomycin C (Sigma) was added to stop cell division; at the same time a scratch was made using a plastic pipette tip. At 24, 48, 72 h post scratch, microscopic imaging was performed with a 20× objective (Leica). Each experiment was performed in triplicate.

### Calcium measurements

Cytosolic and submembrane Ca2+ levels were estimated by overexpressing in MPM cells either soluble GCaMP5 Ca2+ reporter or membrane targeted LCK-GCaMP5 Ca^2+^ reporter (KCNMA1), respectively [66]. Cells were co-transfected with DNAs coding for reporters and microRNA mimic or siRNA using Lipofectamine 2000 (Life Technologies). Coverslips with transfected cells were placed into glass bottom Petri dishes (MatTek Corporation, Ashland, MA, USA) in 4 mM K^+^ buffer (150 mM NaCl, 4 mM KCl, 2 mM MgCl_2_, 10 mM Glucose, 10 mM HEPES, 2 mM CaCl_2_). Images of cells were captured using an Eclipse TiE fluorescence microscope (Nikon), Plan Apo VC 60× water-immersion objective (Nikon, numerical aperture 1.2), pE-2 CoolLED excitation light source (CooLED, Yorktwon Height, NY, USA) and NIS-elements software (Version 4.0; Nikon). To analyze depolarization-induced Ca^2+^ influx, 90 mM K^+^ buffer (64 mM NaCl, 90 mM KCl, 2 mM MgCl_2_, 10 mM Glucose, 10 mM HEPES, 2 mM CaCl_2_) was added to cells transfected with LCK-GCaMP5. Images of transfected cells were captured every 0.5 s during the treatment. Fluorescence intensity of the reporter signals was quantified in manually outlined cells using ImageJ (National Institutes of Health) as described [67].

### Statistical analyses

Differential microarray expression analysis was performed using GeneSpring v12.0 using unpaired *t*-tests and candidates selected on the basis of statistical significance (*P* < 0.05) as previously described [19]. Correlation analyses were performed using Pearson-correlation tests and the average expression across all microarray probes for each candidate gene. Group comparisons, correlations and associations were performed using SPSS statistical software and two tailed Mann-Whitney U-tests. A *P*-value less than 0.05 was considered statistically significant. Pathway enrichment analyses were based on KEGG pathways (Partek Genome Suite v6.4).

## Abbreviations

*ALCAM*, activated leukocyte cell adhesion molecule, [GenBank:214, Ensembl:ENSG00000170017; HPRD:03389; MIM:601662; Vega:OTTHUMG00000159192]; *CRI1*, EP300 interacting inhibitor of differentiation 1, [GenBank:23741, Ensembl:ENSG00000255302; HPRD:06907; MIM:605894; Vega:OTTHUMG00000165911]; FFPE, formalin-fixed paraffin embedded; GSEA, Gene Set Enrichment Analysis; *GSN*, gelsolin, [Genbank:2934, Ensembl:ENSG00000148180; HPRD:00674; MIM:137350; Vega:OTTHUMG00000020584,]; KCa1.1, calcium-activated potassium channel 1, subunit alpha 1 protein; *KCNMA1*, calcium-activated potassium channel 1, subunit alpha 1 gene [Gene ID:3778, Ensembl:ENSG00000156113; HPRD:15967; MIM:600150; Vega:OTTHUMG00000018543]; KEGG, Kyoto Encyclopedia of Genes and Genomes; *MMP14*, matrix metallopeptidase 14 (membrane inserted), [GenBank:4323, Ensembl:ENSG00000157227; HPRD:02856; MIM:600754; Vega:OTTHUMG00000028704]; MPM, Malignant Pleural Mesothelioma; *NME2*, NME/NM23 nucleoside diphosphate kinase 2, [GenBank: 4831, Ensembl:ENSG00000011052; Ensembl:ENSG00000243678; HPRD:01132; MIM:156491; Vega:OTTHUMG00000154062]; *PDGFC*, platelet derived growth factor C, [Genbank:56034, HPRD:10529; MIM:608452]

## Additional file

Additional file 1: Table S1. Top 20 Enriched microRNA Families Extracted from the Four Gene Expression Datasets. Table S2. Pathway Enrichment Analysis of Gene Targets of the miR-17 family members. Table S3. Primers and TaqMan Assay IDs for RT-qPCR and siRNA and Mimic Sequences. Table S4. Characteristics of Patients Analyzed in Fig. 2h and i. Table S5. Individual *P* values for Fig. 3a, f and g. Figure S1. *TGFBR2* mRNA down regulated followed miR-17-5p transfection. Figure S2. MPM cell viability was not affected by transfection with miR-17-5p mimic or siRNA. Figure S3. Effect of *KCNMA1* down-regulation on cell cycle in MPM cells. Figure S4. KCNMA1 down-regulation and miR-17-5p did not induce MPM cell apoptosis. Figure S5. Migration of MPM cell lines treated with miR-17-5p mimic or *KCNMA1* siRNAs. Figure S6. Invasion of MPM cell lines treated with miR-17-5-5p mimic or *KCNMA1* siRNAs. Figure S7. Paxilline did not sensitize MPM cells to cisplatin or gemcitabine. (DOCX 10063 kb)
