# Supplementary material for: KCa1.1, a calcium-activated potassium channel subunit alpha 1, is targeted by miR-17-5p and modulates cell migration in malignant pleural mesothelioma
Source: Mol Cancer. 2016 Jun 1;15:44. doi: 10.1186/s12943-016-0529-z (PMC4888473; doi:10.1186/s12943-016-0529-z)
Supplement: Additional file 1: Table S1. — Top 20 Enriched microRNA Families Extracted from the Four Gene Expression Datasets. Table S2. Pathway Enrichment Analysis of Gene Targets of the miR-17 family members. Table S3. Primers and TaqMan Assay IDs for RT-qPCR and siRNA and Mimic Sequences. Table S4. Characteristics of Patients Analyzed in Fig. 2h and i. Table S5. Individual P values for Fig. 3a, f and g. Figure S1. TGFBR2 mRNA down regulated followed miR-17-5p transfection. Figure S2. MPM cell viability was not affected by transfection with miR-17-5p mimic or siRNA. Figure S3. Effect of KCNMA1 down-regulation on cell cycle in MPM cells. Figure S4. KCNMA1 down-regulation and miR-17-5p did not induce MPM cell apoptosis. Figure S5. Migration of MPM cell lines treated with miR-17-5p mimic or KCNMA1 siRNAs. Figure S6. Invasion of MPM cell lines treated with miR-17-5-5p mimic or KCNMA1 siRNAs. Figure S7. Paxilline did not sensitize MPM cells to cisplatin or gemcitabine. (DOCX 10063 kb) [file 12943_2016_529_MOESM1_ESM.docx]

**SUPPLEMENTARY MATERIALS AND METHODS**

**Cell cycle analysis**

To assess the effects of *KCNMA1* knockdown on the cell cycle, MPM cells were seeded in 6-well plates at a density of 2x10^5^ cells/well. After incubation for 48 h, cells were harvested and washed 3 times with PBS. Cells were fixed overnight in 70 % ethanol. Fixing solution was removed and cells were treated with 0.01 % RNase (10 mg/mL, Sigma), 0.05 % propidium iodide in PBS for 30 min at 37 ˚C in the dark. The cell cycle distribution was determined on an Accuri C6 flow cytometer (BD Biosciences, location) within 30 minutes. The flow cytometer was calibrated using 6- and 8-peak fluorescent bead mixtures provided by the manufacturer, and according to the manufacturer’s instructions (Accuri, Ann Arbor, MI, USA). The flow cytometer was operated at the Slow Flow Rate setting (14 μL sample/minute), and data acquisition for a single sample typically required 3-5 min. For each sample 10,000 events were counted and analyzed using FlowJo software (Ashland, OR, USA). Each experiment was performed in triplicate.

**Agarose spot invasion assay**

The invasive ability of MPM cells transfected with *KCNMA1*-specific siRNA or miR-17-5p mimic was measured using a modified agarose spot assay as described previously [1, 2]. At 48 h post seeding, microscopic imaging was performed with a 20x objective, and for each spot we recorded the field that contained the highest apparent number of motile cells penetrating furthest underneath the agarose spot.

**Table S1: Top 20 Enriched microRNA families* extracted from the four gene expression datasets.**

| **WRIGHT** |  | **FDR** | **CRISPI** |  | **FDR** | **SURAOKAR** |  | **FDR** | **GORDON** |  | **FDR** |
| --- | --- | --- | --- | --- | --- | --- | --- | --- | --- | --- | --- |
| **Gene Set Name** | **P-value** | **q-value** | **Gene Set Name** | **P-value** | **q-value** | **Gene Set Name** | **P-value** | **q-value** | **Gene Set Name** | **P-value** | **q-value** |
| miR-124a | 4.62E-16 | 1.02E-13 | miR-17, miR-20a/b, miR-106a/b, miR-519d | 1.65E-12 | 3.32 e^-10^ | miR-124a | 7.13E-29 | 1.58E-26 | miR-181a/b/c/d | 2.48E-11 | 5.49E-09 |
| miR-519a/b/c | 1.70E-15 | 1.88E-13 | miR-27a/b | 3.01E-12 | 3.32 e^-10^ | miR-30a-e | 8.79E-20 | 9.71E-18 | miR-25, miR-32, miR-92, miR-363, miR-367 | 4.88E-09 | 0.000000539 |
| miR-506 | 3.54E-15 | 2.61E-13 | miR-212, miR-132 | 1.03 e^-10^ | 7.56 e^-9^ | miR-182 | 4.24E-18 | 3.13E-16 | miR-199a | 1.48E-08 | 0.00000109 |
| miR-181a/b/c/d | 3.43E-14 | 1.90E-12 | miR-520g/h | 1.89 e^-9^ | 1.04 e^-7^ | miR-23a/b | 7.04E-18 | 3.89E-16 | miR-96 | 2.25E-08 | 0.00000124 |
| miR-30a-e | 5.07E-14 | 2.24E-12 | miR-30a/e-3p | 1.76 e^-8^ | 7.79 e^-7^ | miR-19a/b | 9.64E-18 | 4.26E-16 | miR-493 | 3.68E-08 | 0.00000163 |
| miR-9 | 1.10E-13 | 4.03E-12 | miR-374 | 2.16 e^-8^ | 7.95 e^-7^ | miR-506 | 1.91E-17 | 7.03E-16 | miR-23a/b | 6.99E-08 | 0.00000239 |
| miR-520a-e(-5p) | 1.93E-13 | 6.09E-12 | miR-128a/b | 3.24 e^-8^ | 1.02 e^-6^ | miR-493 | 9.21E-17 | 2.91E-15 | miR-200b/c, miR-429 | 7.57E-08 | 0.00000239 |
| miR-19a/b | 1.41E-12 | 3.90E-11 | miR-96 | 5.06 e^-8^ | 1.4 e^-6^ | miR-96 | 1.07E-16 | 2.96E-15 | miR-27a/b | 8.92E-08 | 0.00000246 |
| miR-15a/b, miR-16, miR-195, miR-424, miR-497 | 8.86E-12 | 2.01E-10 | miR-519a/b/c | 5.71 e^-8^ | 1.4 e^-6^ | miR-15a/b, miR-16, miR-195, miR-424, miR-497 | 1.27E-16 | 3.12E-15 | miR-30a-e | 1.01E-07 | 0.00000248 |
| miR-200b/c, miR-429 | 9.08E-12 | 2.01E-10 | miR-30a-e | 1.23 e^-7^ | 2.7 e^-6^ | miR-519a/b/c | 1.59E-16 | 3.52E-15 | miR-93, miR-302a-d, miR-372/373, miR-520a-e(-3p) | 1.23E-07 | 0.00000252 |
| miR-17, miR-20a/b, miR-106a/b, miR-519d | 2.01E-11 | 4.04E-10 | miR-144 | 1.34 e^-7^ | 2.7 e^-6^ | miR-218 | 2.32E-16 | 4.66E-15 | miR-26a/b | 1.26E-07 | 0.00000252 |
| miR-29a-c | 8.30E-11 | 1.53E-09 | miR-506 | 2.78 e^-7^ | 5.12 e^-6^ | miR-101 | 1.78E-14 | 3.05E-13 | miR-182 | 3.78E-07 | 0.00000696 |
| miR-130a/b, miR-301 | 2.02E-10 | 3.44E-09 | miR-101 | 3.09 e^-7^ | 5.25 e^-6^ | miR-200b/c, miR-429 | 1.79E-14 | 3.05E-13 | miR-17, miR-20a/b, miR-106a/b, miR-519d | 6.68E-07 | 0.0000114 |
| miR-101 | 4.53E-10 | 7.15E-09 | miR-409-3p | 3.6 e^-7^ | 5.68 e^-6^ | miR-27a/b | 2.82E-14 | 4.45E-13 | miR-506 | 1.07E-06 | 0.0000168 |
| miR-135a/b | 1.02E-09 | 1.51E-08 | miR-138 | 5.27 e^-7^ | 7.77 e^-6^ | miR-203 | 3.53E-14 | 5.21E-13 | miR-139 | 1.95E-06 | 0.0000287 |
| miR-93, miR-302a-d, miR-372/373, miR-520a-e(-3p) | 1.86E-09 | 2.57E-08 | miR-22 | 6.96 e^-7^ | 9.61 e^-6^ | miR-130a/b, miR-301 | 1.03E-13 | 1.42E-12 | miR-144 | 2.30E-06 | 0.0000314 |
| miR-141, miR-200a | 2.27E-09 | 2.95E-08 | miR-182 | 8.11 e^-7^ | 9.99 e^-6^ | miR-17, miR-20a/b, miR-106a/b, miR-519d | 3.59E-13 | 4.67E-12 | miR-494 | 2.47E-06 | 0.0000314 |
| let-7a/b/c/d/e/f/g/i, miR-98 | 4.06E-09 | 4.72E-08 | miR-34a/c, miR-449 | 8.14 e^-7^ | 9.99 e^-6^ | miR-181a/b/c/d | 8.19E-13 | 1.01E-11 | miR-203 | 2.59E-06 | 0.0000314 |
| miR-330 | 3.89E-09 | 4.72E-08 | miR-488 | 8.77 e^-7^ | 1.02 e^-5^ | miR-154, miR-487 | 5.98E-12 | 6.96E-11 | miR-223 | 2.74E-06 | 0.0000314 |
| miR-103, miR-107 | 6.70E-09 | 7.21E-08 | miR-142-5p | 1.11 e^-6^ | 1.21 e^-5^ | miR-29a-c | 1.45E-11 | 1.6E-10 | miR-520a-e(-5p) | 2.84E-06 | 0.0000314 |

***** As described in Fig. 1 using Gene Set Enrichment Analysis; shaded boxes represent the microRNA families commonly enriched in all datasets.

**Table S2: Pathway Enrichment Analysis of Gene Targets* of the miR-17 Family Members.**

| **Pathway Name** | **Enrich-ment Score** | **Enrichment p-value** | **% genes in pathway that are present** | **# genes in list, in pathway** | **KEGG Path-way ID** |
| --- | --- | --- | --- | --- | --- |
| Endocytosis | 9.28788 | 9.25E-05 | 2.83688 | 24 | 232 |
| Axon guidance | 7.72536 | 0.000441487 | 3.04659 | 17 | 151 |
| MAPK signaling | 6.76363 | 0.00115503 | 2.23821 | 28 | 261 |
| Pancreatic cancer | 5.97754 | 0.00253505 | 3.10881 | 12 | 249 |
| Circadian rhythm | 5.56409 | 0.00383305 | 4.83871 | 6 | 131 |
| Hepatitis B | 5.41736 | 0.00443883 | 2.44957 | 17 | 89 |
| Prostate cancer | 5.37944 | 0.00461041 | 2.76596 | 13 | 234 |
| Neurotrophin signaling | 5.32326 | 0.00487683 | 2.5641 | 15 | 50 |
| Renal cell carcinoma | 5.06674 | 0.00630293 | 3.0581 | 10 | 192 |
| ErbB signaling | 4.93199 | 0.00721211 | 2.72109 | 12 | 269 |
| Focal adhesion | 4.91313 | 0.00734946 | 2.22743 | 19 | 188 |
| Bladder cancer | 4.88023 | 0.00759527 | 3.40426 | 8 | 171 |
| Glioma | 4.86436 | 0.00771677 | 2.96736 | 10 | 182 |
| Regulation of actin cytoskeleton | 4.84241 | 0.00788802 | 2.21187 | 19 | 139 |
| Hepatitis C | 4.75531 | 0.00860586 | 2.40385 | 15 | 194 |
| TNF signaling | 4.65984 | 0.00946795 | 2.37718 | 15 | 243 |
| Chronic myeloid leukemia | 4.62135 | 0.00983953 | 2.72277 | 11 | 69 |
| FoxO signaling | 4.40994 | 0.0121559 | 2.30769 | 15 | 86 |
| Melanoma | 4.10599 | 0.0164737 | 2.63852 | 10 | 27 |
| Prolactin signaling | 3.71527 | 0.0243488 | 2.47525 | 10 | 9 |
| Non-small cell lung cancer | 3.53683 | 0.0291055 | 2.65781 | 8 | 30 |
| TGF-beta signaling | 3.51994 | 0.0296011 | 2.50696 | 9 | 7 |
| HTLV-I infection | 3.44629 | 0.0318635 | 1.85512 | 21 | 190 |
| Ras signaling | 3.36306 | 0.034629 | 1.91489 | 18 | 265 |
| Colorectal cancer | 3.23097 | 0.0395193 | 2.5 | 8 | 111 |
| PI3K-Akt signaling | 3.21181 | 0.0402837 | 1.73611 | 25 | 262 |
| Dorso-ventral axis formation | 3.16795 | 0.0420899 | 3.66972 | 4 | 260 |
| Insulin signaling | 3.04641 | 0.0475291 | 2.06897 | 12 | 16 |
| p53 signaling | 3.01547 | 0.0490226 | 2.53623 | 7 | 32 |
| Vasopressin-regulated water reabsorption | 3.00164 | 0.0497054 | 3.01205 | 5 | 57 |
| Glycosphingolipid biosynthesis - ganglio series | 2.99898 | 0.0498381 | 4.34783 | 3 | 241 |
|  |  |  |  |  |  |

*****miRDB – MicroRNA Target Prediction And Functional Study Database ([www.mirdb.org](http://www.mirdb.org)) was utilized to capture gene targets for miR-17 family members.

**Table S3: Primers and TaqMan Assay IDs for RT-qPCR, and siRNA and Mimic Sequences.**

| ***microRNA*** | ***TaqMan assay ID*** |  |
| --- | --- | --- |
| hsa-miR-17-5p | 000393 |  |
| hsa-miR-19b-3p | 000396 |  |
| hsa-miR-20a-5p | 000580 |  |
| hsa-miR-106a-5p | 002169 |  |
| hsa-miR-30e-5p | 002223 |  |
| RNU6B | 001093 |  |
| ***mRNA target*** | ***Forward Primer (5’-3’)*** | ***Reverse Primer (5’-3’)*** |
| *KCNMA1* | TCGGAGTCTTGCAGGCTAAT | GTTGTGATGGATGGTTGACG |
| *TGFBR2* | GGGAAATGACATCTCGCTGTA | CACCTTGGAACCAAATGGAG |
| 18S | GCCGCTAGAGGTGAAATTCTTG | CATTCTTGGCAAATGCTTTCG |
| ***siRNA/ mimic*** | ***Sense (5’-3’)*** | ***Antisense (5’-3’)*** |
| *KCNMA1*.1 | GACACUGACUGGCAGAGUCCUGGUUGU | ACAACCAGGACUCUGCCAGUCAGUGUC |
| *KCNMA1*.2 | GCCUUCGUGGGUCUGUCCUUCCCUACU | AGUAGGGAAGGACAGACCCACGAAGGC |
| Ctrl siRNA | CCACACGAGUCUUACCAAGUUGCUU | AAGCAACUUGGUAAGACUCGUGUGG |
| miR-17-5p | CUACCUGCACUGUAAGCACUUUG | CAAAGUGCUUACAGUGCAGGUAG |

**Table S4: Characteristics of Patients Analyzed in Fig. 2H & 2I**

| **Patient*** | **Gender#** | **Age** | **MPM**  **Histological Subtype** |
| --- | --- | --- | --- |
| 1 | Male | 68 | Biphasic |
| 2 | Male | 67 | Epithelioid |
| 3 | Male | 65 | Epithelioid |
| 4 | Male | 76 | Epithelioid |
| 5 | Male | 69 | Biphasic |
| 6 | Male | 71 | Epithelioid |
| 7 | Male | 70 | Epithelioid |
| 8 | Male | 68 | Biphasic |
| 9 | Male | 65 | Epithelioid |
| 10 | Male | 89 | Epithelioid |
| 11 | Female | 70 | Epithelioid |
| 12 | Male | 61 | Epithelioid |
| 13 | Female | 63 | Biphasic |
| 14 | Male | 72 | Epithelioid |
| 15 | Male | 72 | Biphasic |
| 16 | Male | 52 | Epithelioid |

* Patients are numbered according to the heat map in Fig. 2H & 2I, # Patients were selected based on matched age and gender to the available normal pleural samples.

**Table S5: Individual *P* values of Fig 3A, F & G.**

**1) Individual *P* values for Fig 3A.**

|  |  |  |  |  |  |
| --- | --- | --- | --- | --- | --- |
|  | *P* value of control treated versus | | |  |  |
| *KCNMA1* | siRNA 1 | siRNA 2 | miR-17 |  |  |
| H2452 | 2.56718E-06 | 0.000154988 | 0.019 |  |  |
| H28 | 0.005765 | 1.85714E-06 | 0.025 |  |  |
| MSTO | 0.000563384 | 1.06383E-05 | 0.152 |  |  |
| MM05 | 5.03327E-05 | 1.78716E-05 | 0.005 |  |  |
| Ren | 0.026 | 2.56273E-07 | 0.004 |  |  |
|  |  |  |  |  |  |

**2) Individual *P* values for Fig 3F.**

|  | *P* value of untreated control versus | | | |  |
| --- | --- | --- | --- | --- | --- |
|  | 6.25 μM | 12.5 μM | 25 μM | 100 μM |  |
| H2452 | 0.88 | 0.05 | 3.26092E-05 | 1.13966E-06 |  |
| H28 | 0.28 | 0.85 | 0.000821949 | 5.90562E-08 |  |
| MSTO-H211 | 0.06 | 0.05 | 0.634 | 1.28371E-06 |  |
| MM05 | 0.77 | 0.48 | 0.028 | 5.94933E-05 |  |
| Ren | 0.76 | 0.06 | 0.008 | 2.03278E-05 |  |
|  |  |  |  |  |  |

**3) Individual *P* values for Fig 3G.**

|  | *P* value of control vs Paxilline |
| --- | --- |
| H2452 | 3.9703E-08 |
| H28 | 2.91858E-07 |
| MSTO | 6.71449E-07 |
| Ren | 6.1894E-07 |
| MM05 | 7.2378E-07 |

**Figure S1:** ***TGFBR2* mRNA is down-regulated following KCNMA1-specific siRNA and miR-17-5p mimic transfection.**

**
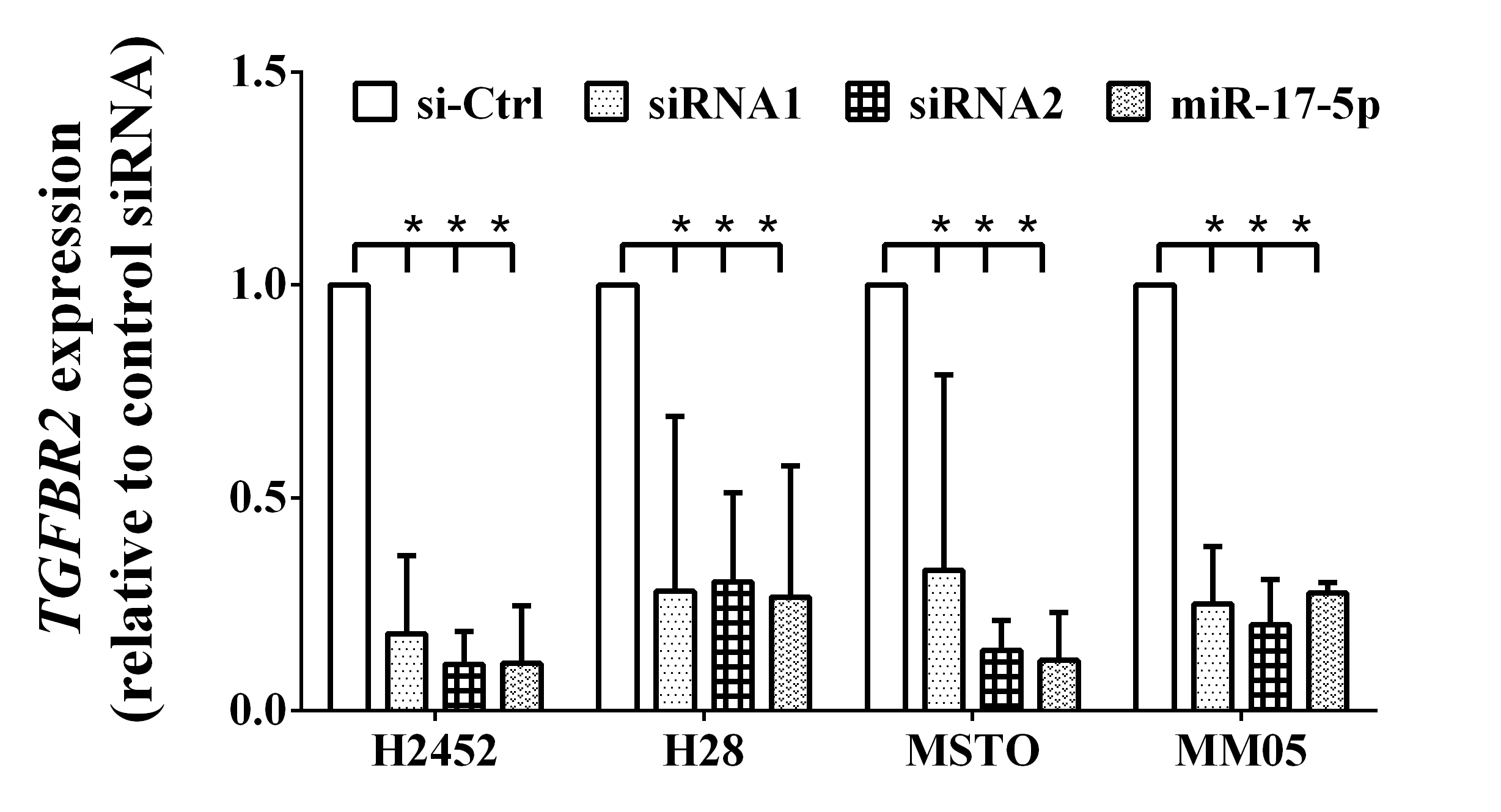
**

**Figure S1: *TGFBR2* mRNA is down-regulated following *KCNMA1*-specific siRNA and miR-17-5p mimic transfection.** RT-qPCR showed *TGFBR2* down-regulated in a panel of MPM cell lines following *KCNMA1*-specific siRNA and miR-17-5p mimic transfection (* indicates *P* < 0.01). Individual *P* value for each transfection is presented in this Table.

| ***TGFBR2*** | *P* value of control treated versus | | |
| --- | --- | --- | --- |
|  | siRNA 1 | siRNA 2 | miR-17-5p |
| H2452 | 0.00152895 | 3.64419E-05 | 0.000342977 |
| H28 | 0.0385961 | 0.00454176 | 0.0146692 |
| MSTO | 0.0649869 | 2.80555E-05 | 0.000170283 |
| MM05 | 0.000635502 | 0.000198585 | 9.72218E-07 |

Note: siRNA1 = *KCNMA1*.1 siRNA, siRNA2 = *KCNMA1*.2 siRNA, si-Ctrl = siRNA control.

**Figure S2: MPM cell viability was not affected by transfection with miR-17-5p mimic or *KCNMA1*-specific siRNA**

**
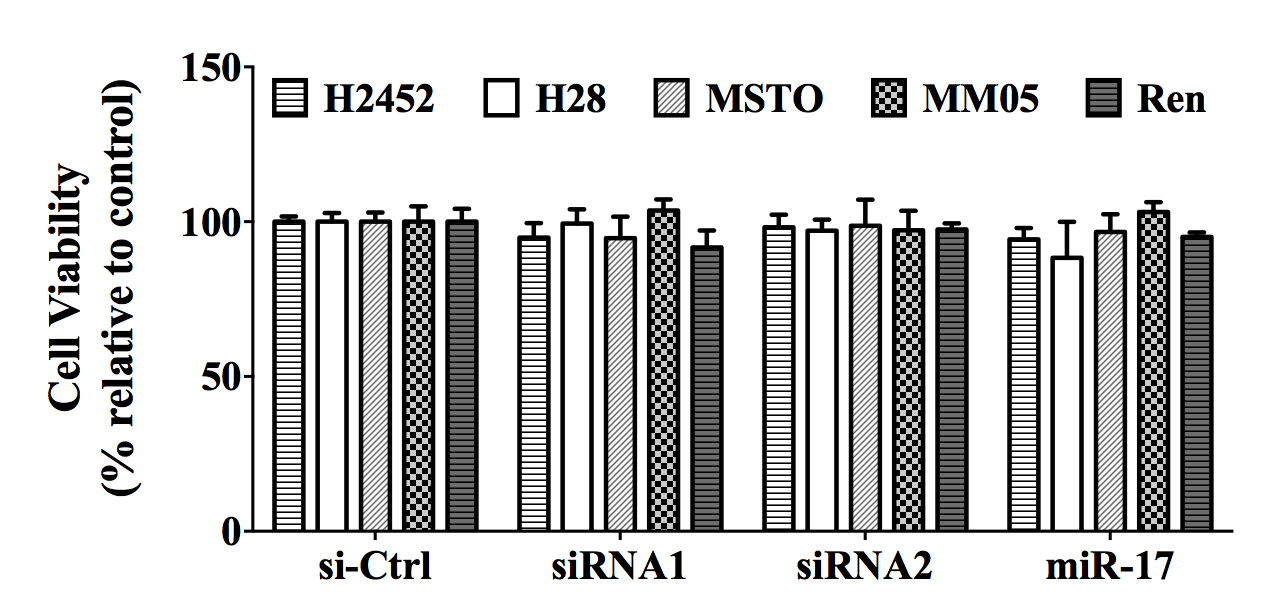
**

**Figure S2: MPM cell viability was not affected by transfection with miR-17-5p mimic or *KCNMA1*-specific siRNA.** MPM cells (H2452, H28, MSTO, MM05 and Ren) were transfected with miR-17-5p mimic or siRNA specific for *KCNMA1*, at 10 nM of either mimic or siRNA did not inhibit cell viability at 72 hr post-transfection.

**Figure S3: Effect of *KCNMA1* down-regulation on cell cycle in MPM cells.**

**
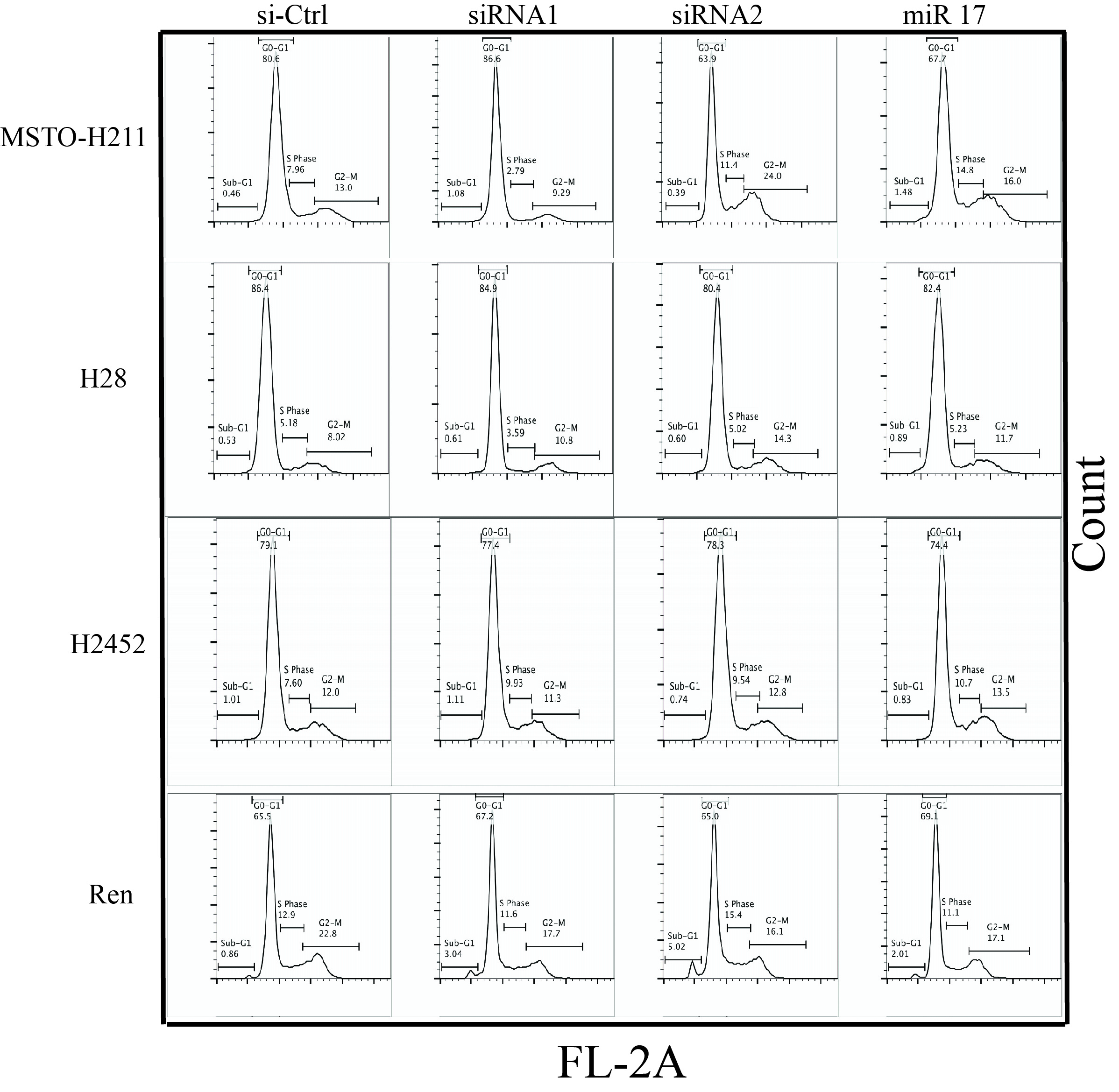
Figure S3: Effect of *KCNMA1* down-regulation on cell cycle in MPM cells.** Transfection with *KCNMA1*-specific siRNA or miR-17-5p mimic did not cause cell cycle arrest in MSTO, H28, H2452 and Ren. (Representative histograms from three individual experiments were shown). Note: MSTO = MSTO-H211.

**Figure S4: *KCNMA1* down-regulation and miR-17-5p did not induce MPM cell apoptosis.**


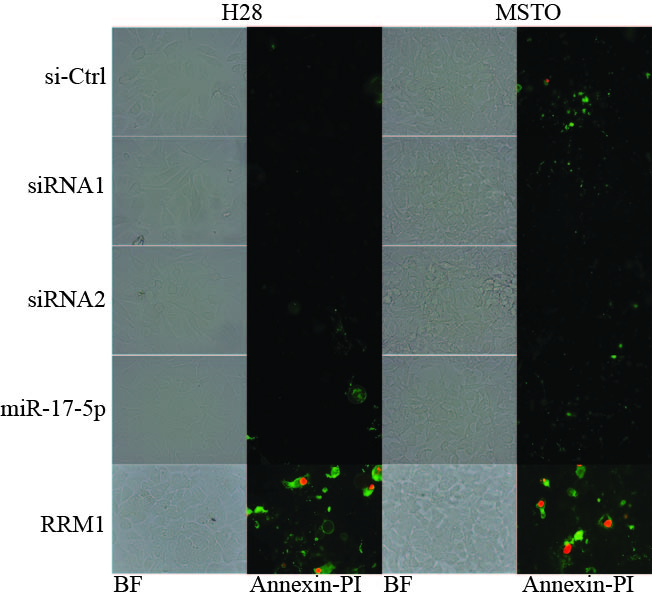


**Figure S4: *KCNMA1* down-regulation and miR-17-5p did not induce MPM cell apoptosis.** Transfecting H28 and MSTO with miR-17-5p mimic or *KCNMA1*-specific siRNA did not induce cell apoptosis. RRM1 was a positive control of apoptosis. Green indicates presence of early apoptosis (annexin V), red indicates cell death (propidium iodide staining) and presence of both red and green fluorescence indicates necrosis (Axio imager.M2, Zeiss with a 40x objective).

**Figure S5: Migration of MPM cell lines treated with miR-17-5p mimic or *KCNMA1* siRNAs.**

**
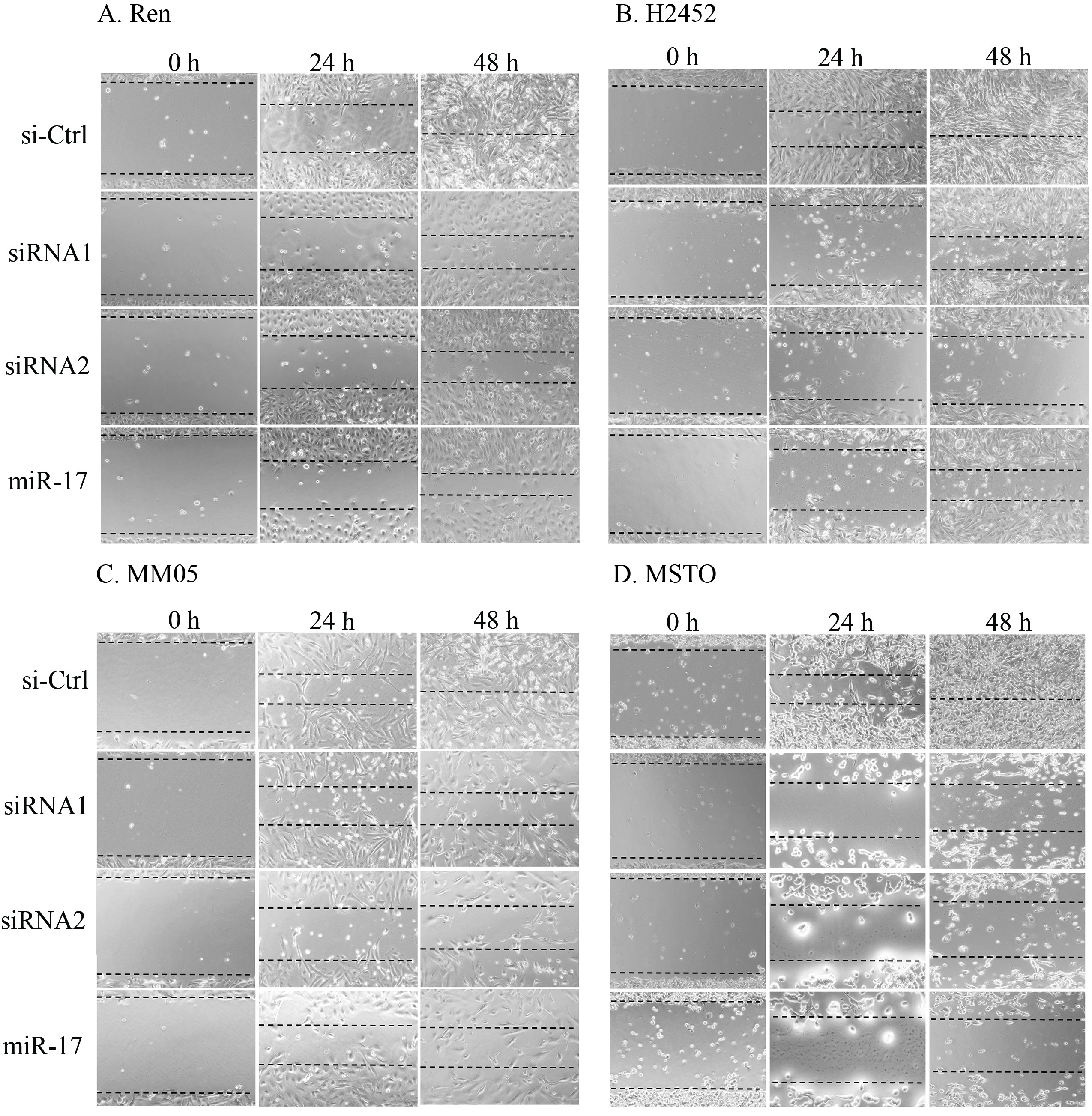
**

**Figure S5: Migration of MPM cell lines treated with miR-17-5p mimic or *KCNMA1* siRNAs.** Transfection of *KCNMA1­*-specific siRNA or miR-17-5p mimic suppressed MPM cell migration in MPM cell lines; Ren (A), H2452 (B), MM05 (C) and MSTO (D). siRNA1 = *KCNMA1*.1 siRNA, siRNA2 = *KCNMA1*.2 siRNA, si-Ctrl = siRNA control, MSTO = MSTO-H211.**Figure S6: Invasion of MPM cell lines treated with miR-17-5p mimic or *KCNMA1* siRNAs.**

**
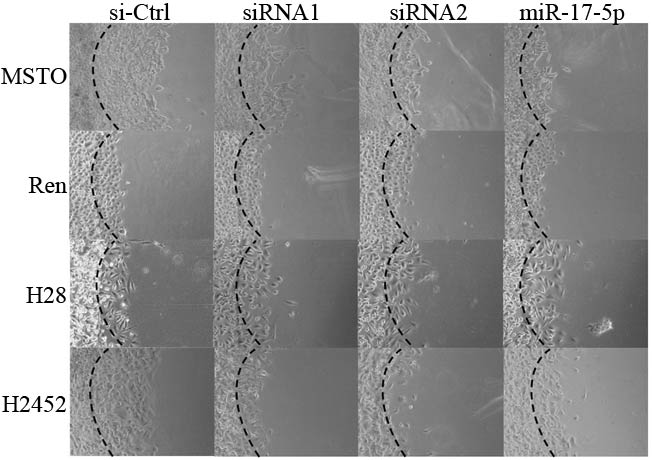
**

**Figure S6: Invasion of MPM cell lines treated with miR-17-5p mimic or *KCNMA1* siRNAs.** Transfection with *KCNMA1-*specific siRNAs or miR-17-5p mimic suppressed MPM cell invasion in MSTO, REN, H28 and H2452. Dotted line shows the border of agarose spot. A modified agarose spots assay was used [1, 2].

**Figure S7: Paxilline did not sensitize MPM cells to cisplatin or gemcitabine.**

**
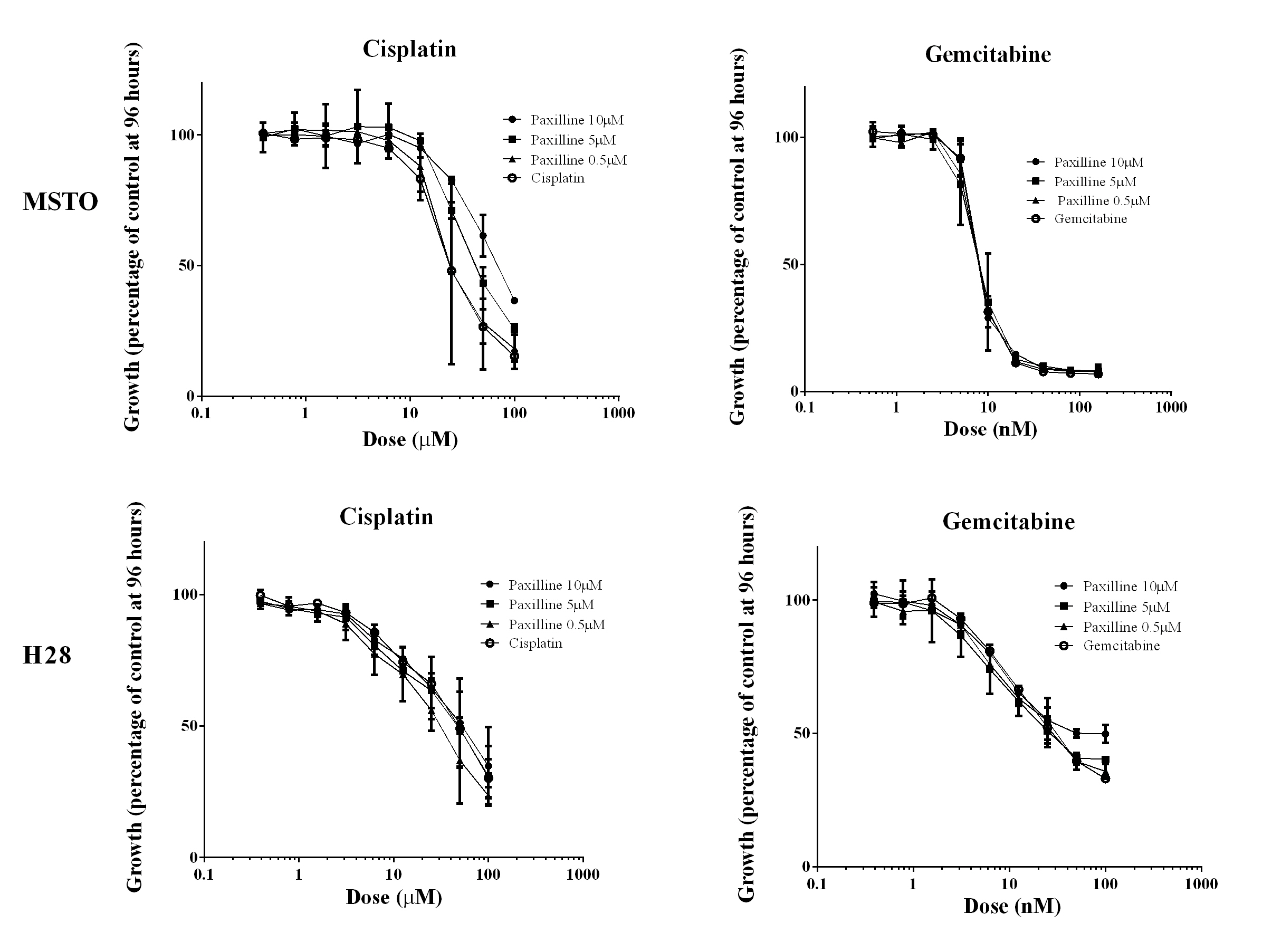
**

**Figure S7: Paxilline did not sensitize MPM cells to cisplatin or gemcitabine.** Different dosage of paxilline (0.5 μM, 5 μM and 10 μM) applied to H28 and MSTO respectively did not sensitize these cell lines to cisplatin or gemcitabine. Percentage of viable cells was plotted with mean ± SD (based on three biological replicates and within each, three technical replicates was carried out, thus n = 9 data points). MSTO = MSTO-H211.

**Reference:**

1. Cheng NC, van Zandwijk N, Reid G: **Cilengitide inhibits attachment and invasion of malignant pleural mesothelioma cells through antagonism of integrins alphavbeta3 and alphavbeta5.** *PLoS One* 2014, **9:**e90374.

2. Cheng YY, Kirschner MB, Cheng NC, Gattani S, Klebe S, Edelman JJ, Vallely MP, McCaughan BC, Jin HC, van Zandwijk N, Reid G: **ZIC1 is silenced and has tumor suppressor function in malignant pleural mesothelioma.** *J Thorac Oncol* 2013, **8:**1317-1328.
